# Targeting HDAC6 for chemotherapy‐induced neurotoxicity and Alzheimer’s disease

**DOI:** 10.1002/alz.087772

**Published:** 2025-01-03

**Authors:** Jiacheng Ma, Sunil Goodwani, Morgan McReynolds, Claudia H Huichalaf, Virginie Buggia‐Prevot, Paul Acton, Annemieke Kavelaars, Cobi J Heijnen, William J Ray

**Affiliations:** ^1^ The Neurodegeneration Consortium, UT MD Anderson Cancer Center, Houston, TX USA; ^2^ MD Anderson Cancer Center, HOUSTON, TX USA

## Abstract

**Background:**

Chemotherapy‐induced cognitive impairment (CICI) is a commonly reported neurotoxic side effect of chemotherapy, occurring in up to 75% cancer patients. Connections between chemo‐treatment and increased risk of dementia have been reported. Mechanistically, chemotherapy treatment contributes to an accelerated aging phenotype in the brain through induction of pathogenic tau, disruption of neuronal integrity, reactive gliosis and neuroinflammation. Our work identified histone deacetylase 6 (HDAC6) as a potential therapeutic target against CICI. The primary substrates of HDAC6 include non‐histone cytoplasmic substrates, such as the microtubule protein α‐tubulin. Inhibition of HDAC6 has been shown to improve axonal transport of mitochondria and preserve axonal bioenergetics, the disruption of which are key factors contributing to cognitive impairment associated with chemotherapy and Alzheimer’s disease (AD). Therefore, inhibition of HDAC6 may provide benefits against chemotherapy‐induced neurotoxicity and Alzheimer’s disease.

**Method:**

CICI was established in mice with two cycles of cisplatin treatment. A brain‐penetrant HDAC6 inhibitor was then administered for two weeks. Behavioral testing, immunohistochemistry and biochemical approaches were taken to determine the effect of HDAC6 inhibition on cognitive function, synaptic mitochondrial function, synaptic integrity and tau pathology. To determine the therapeutic potential of targeting HDAC6 for AD, we crossed the Tg4510 tauopathy mouse model with HDAC6 knockout mice and evaluated behavioral changes and markers of neurodegeneration including NeuN staining and plasma neurofilament‐light.

**Result:**

HDAC6 inhibition normalizes cisplatin‐induced cognitive impairments, restores microtubule stability and reverses tau phosphorylation, leading to normalization of synaptic mitochondrial function and synaptic integrity in CICI. These data suggest that short‐term treatment with a brain‐penetrant HDAC6 inhibitor is sufficient to achieve prolonged reversal of functional deficits induced by chemotherapeutics. In the context of AD, short‐term treatment with the HDAC6 inhibitor did not provide benefits in the Tg4510 tauopathy mouse model. However, knockout of HDAC6 in Tg4510 mice reverses the hyperactive behavioral phenotype, preserves neuronal integrity and reduces plasma neurofilament‐light level up to 6‐month of age.

**Conclusion:**

HDAC6 inhibition has great therapeutic potential for both chemotherapy‐induced neurotoxicity and AD.

